# Late Ligation of the Cord

**Published:** 1887-03

**Authors:** J. W. Keene


THE BUFFALO MEDICAL AND SURGICAL JOURNAL

VOL. XXVI. MARCH, 1887. No. 8

### Original Communications.

### *LATE LIGATION OF THE CORD.**

By J. W. KEENE, A. M., M. D.

It has been my fortune several times, when ligating the cord immediately after the birth of the child and before cessation of pulsation in the umbilical arteries, to observe most manifest evidences of discomfort and suffering on the part of the new-born. It has been my custom obeying the teaching of a most conservative obstetrician, the late Dr. Buckingham of Boston, to wait for the pulsations to cease before tying. But many cases occur where a real or fancied necessity exists for disregarding this rule; and in such cases the observation above noted has obtained in every instance, unless the child was asphyxiated or otherwise unable to manifest its suffering. Presumably this is the general experience of the profession, although not mentioned in any book consulted.

As to the propriety of tying the cord at all, the warfare has been hot and protracted, but at present in most civilized nations, and among savage tribes as well, the custom of tying prevails. Indeed, from the earliest times and among the most primitive

*Read before the Buffalo Obstetrical Society.

people, as well as among those more advanced in civilization, tying the cord has been practiced as a readily-suggested means of preventing or arresting hemorrhage. Those astute physicians who base so many excellent theories on the incontestable fact that man is an animal, and, therefore, will or should behave under similar conditions just as a frog or a rabbit does, have denied that ligation is necessary. Cats and rabbits, cows and mares do not ligate the cord, it must be confessed, but the cord torn by the teeth or broken by the fall does not bleed. In street births, and similar accidents in which the cord is pulled asunder by the weight of the falling child, no hemorrhage occurs, and for the same reason. Among some savage races, too, the cord, sawed by a shell, a flint knife, a piece of split bamboo or a rough leaf-stalk, does not bleed even when not tied. Still, the custom of tying is very generally practiced.

Having decided, then, that it is better to tie than not to tie, another opportunity for a contest presents itself—a slender one to be sure, but, nevertheless, a chance for dispute not to be ignored by the disputations. Shall we tie and cut, or cut and tie? Pajot taught that the latter is the proper sequence, even allowing some blood to escape. Cazeaux cuts and then pinches the end to prevent hemorrhage till the ligature can be applied. Recent authors are quite unanimously in favor of tying before cutting. Asphyxia and accidents incident to delivery may render it advisable or necessary to reverse the order. All have witnessed the beneficial effects of allowing a teaspoonful of blood to escape from the funis when asphyxia has induced an apoplectic condition in the child.

Is it necessary to ligate the placental end of the cord? Here again is a chance for opinions to differ, and the opportunity offered has not been neglected. The warmest advocates of the second ligature hold that cleanliness, if no other reason, demands its use. It seems to me that the ounce or two of blood escaping from the placental end of the cord is hardly a material addition to the abundance of blood and scrum, meconium and fæces which invariably and inevitably marks the gory scene of the obstetrician's battlefield, from which he retires victorious or may be baffled or defeated, but seldom in disgrace or without glory. Certainly, in twin deliveries the second ligation is necessary or at least advisable. In this the authorities are quite at one. When, therefore, we have all become infallible in diagnosticating twin births, we may safely omit the second ligature except in such cases. Till that time arrives, it will be better to apply the second ligature. It undoubtedly aids in the separation of the placenta. At the same time, it increases the bulk which must pass the rapidly-contracting cervix when detached. This, however, is probably a theoretical rather than a practical disadvantage, since when once separated, the placenta can empty itself of blood into the cavity of the uterus. It certainly is not necessary when the placenta is detached and lying in the vaginal canal.

When should the ligature be applied? The ancients who were not imbued with the manifold excellences of Credé's manipulation, deferred ligating till the placenta was expelled.

Winkler, 1872, from anatomical considerations alone, advised this course on the ground that by delay a portion of placenta blood would pass to the child, increasing by so much its powers of resistance. He suggested further that this practice might diminish the usual loss of weight observed in the new-born during the first few days of its life. This has been confirmed by the observations of Hoffmeier, Ribémont, Budin and Zweifel.

Budin cites Mauriceau, Clément and Deventer as advocating this plan, but at the same time hastening delivery by artificial means.

In quite a number of cases in which I have adopted it, the plan has proved very satisfactory. One point greatly in its favor is, that the accoucheur is at liberty to follow down the uterus himself instead of delegating that duty to another at the most critical time to the mother during the delivery. But in view of experiments soon to be cited, it may be an injudicious procedure if used in connection with Credé's manipulation.

While it may not be necessary or advisable to defer the application of the ligature till the complete expulsion of the placenta has occurred, the prevailing custom of applying it immediately upon the birth of the child cannot be too strongly deprecated.

A warning cry against precipitate action has again and again been raised.

In 1773, Charles White wrote as follows: "The common method of tying and cutting the navel-string the instant the child is born, is likewise one of those errors in practice that has nothing to plead in its favor but custom. Can it be supposed that this important event, this great change which takes place in the lungs, the heart and the liver from the state of a fœtus kept alive by the umbilical cord to that state when life cannot be carried on without respiration, whereby the lungs must be fully expanded with air, and the whole mass of blood, instead of one-fourth part, be circulated through them, the ductus venosus, foramen ovale, ductus arteriosus, and the umbilical arteries and veins must all be closed and the mode of circulation in the principal vessels entirely altered—is it possible that this wonderful alteration in the human machine should be properly brought about in one instant of time, and at the will of a by-stander? Let us but leave the affair to Nature and watch her operations, and it will soon appear that she stands not in need of our feeble assistance, but will do the work herself at a proper time and in a better manner. In a few minutes the lungs will be gradually expanded, and the great alterations in the heart and blood-vessels will take place. As soon as this is perfectly done, the circulation in the navel-string will cease of itself, and then, if it be cut, no hemorrhage will ensue from either end."

So, too, Braun, after describing the changes from the fœtal to the post-natal circulation, says: "This stupendous process should be taken into consideration in every case of labor, and because of it the cord should never be severed or tied so long as pronounced pulsations can be felt near the navel."

Nägele advised waiting until the pulsations have ceased. Schroeder, Verrier and Porak share this belief. Stoltz recommends waiting until respiration has begun. This view is favored by Hélot of Rouen, and Lusk inclines toward it.

The experiments of Budin, made in 1875 at the suggestion of Tarnier at the Maternité of Paris, are of the greatest interest in this connection. In two series of seventy-five cases, first, where the cord was tied immediately, and second, where it was not tied for several minutes, the amount of blood escaping from the placental end was measured and found to be nearly three ounces less in the second series than in the first. This startling result led him to state that, "to tie and cut the cord directly following delivery, is to prevent the fœtus from drawing 92 grammes (3 ounces) of blood from the placenta, which is equivalent to bleeding an adult to the extent of 1.700 grammes" (60 ounces). If we take Welcker's estimate of the amount of fœtal blood (one-nineteenth of its body weight), the figures assume a yet more appalling proportion. It is probable, however, that Welcker's estimate is much too low. He would allow a child of 7 pounds weight rather less than 6 ounces of blood. The loss of 3 ounces to such an individual could hardly fail to be disastrous. Dalton estimates the quantity of blood at one-eighth the body weight (in adults); this would give the child of 7 pounds 14 ounces of blood. Kirkes' estimate of one-twelfth to one-fourteenth would give the child eight to nine and a third ounces—probably a nearer approximation to the fact than either Welcker's or Dalton's. This seems to be about the estimate on which Budin based his comparison.

These experiments have been abundantly confirmed. Schücking in 1877, and Hélot (of Rouen) at about the same time, modified the experiments of Budin by weighing the child with the cord intact as soon as born and observing the changes till respiration was well established. It was found to gain on the average 53 grammes—(Hélot) 1 4-5 ounces, or 62 grammes; (Schücking) 2 1-5 ounces. At the lowest estimate, then, the child whose maternal connection is severed immediately after birth is deprived of nearly 1-6 of its possible blood supply, or, to resume the comparison of Budin, an amount equal to bleeding an adult weighing 180 pounds 3⅓ pints. It must be borne in mind, too, that the loss is probably greater than this, for a part of the placental blood will have passed to the child before the weight can be determined. Budin and Schücking, who have been foremost in making these experiments, have been led to the same conclusion, viz.: that late ligation ensures an increase of blood to the child. As to the mechanism by which this passage of blood to the fœtus is effected, they hold opposing views. Budin claims that the first inspiration induces a condition of negative pressure—a suction force—which draws the blood into the newly-established pulmonary circulation, and that this thoracic inspiration continues until an equilibrium is established. Schücking holds, on the contrary, that after the first inspiration, this negative pressure ceases, and that further additions to the child's blood supply are caused by the compression exerted by the retraction of the uterus aided intermittently by its contractions. Now, if the latter view is the correct one, the uterine forces so variable in their intensity may, when specially active, drive into the child's circulation a volume of blood in excess of its requirements, and thereby do harm; and especially if these forces are assisted by the hand-exercising compression, according to the method of Credé. Illig, of Kiel, reports a case in which the expressed placenta was forcibly squeezed, driving its contents into the circulation of the child and producing death from over-distention of the heart. It seems possible that the same effect might be produced by practicing uterine compression with the cord intact. That the natural uterine forces alone are capable of producing this disaster, seems to be disproved by the manometric observations made by Ribémont. He found the pressure in the umbilical arteries uniformly greater than in the vein, and that after the pulsation had ceased in the arteries the force of the uterine contractions was insufficient to force the blood to the child through the umbilical vein. That thoracic aspiration exists as an active force, there can be little doubt. The physiological requirements of the child are increased instantaneously by the filling of the lungs with air. This demand must be instantaneously met. We can hardly conceive theoretically of any way in which this can be done so readily as by the exercise of a negative pressure or suction force. On this hypothesis, too, only enough blood to meet the child's needs can pass into its circulation. This seems more consonant with the usual economy of nature than the theory that this increased demand for blood is to be met by the action of the uterine forces, an agency established for an entirely different purpose and so variable and unreliable in accomplishing this *ex-officio* duty, that the child may be overwhelmed by a too forcible and too copious injection of blood into its circulation on the one hand, or, on the other, be obliged to elaborate from its own system, with a certain consumption both of vitality and time, enough blood to meet the increased and instant demand. A simple experiment made by Budin seems conclusive. In a breech presentation, he compressed the cord as far up as possible; on the birth of the child, the vein was distended with blood but instantly emptied with the first inspiration.

As Lusk says: "Thoracic aspiration does, therefore, exist as an operative force." Whatever our view may be as to the mechanism by which this increase of blood reaches the child, we must accept the facts that it will reach the child in some way if we permit it, that it can do the child no harm, and that clinical observation shows that children do better when the cord is ligated late after respiration is established and better still after pulsations have ceased. Lusk sums up the matter as follows:

1. "The cord should not be tied until the child has breathed vigorously a few times. When there is no occasion for haste arising out of the condition of the mother, it is safer to wait until the pulsations of the cord have ceased altogether.

2. "Late ligation is not dangerous to the child. From the excess of blood contained in the fœtal portion of the placenta, the child receives into its system only the amount requisite to supply the needs created by the opening up of the pulmonary circulation.

3. "Until further observations have been made, the practice of employing uterine expression previous to tying the cord is questionable.

4. "In children born pale and anæmic, suffering at birth from syncope, late ligation furnishes an invaluable means of restoring the equilibrium of the fœtal circulation."

In conclusion, gentlemen, let me suggest this thought for your consideration: may we not by judicious delay in tying the cord be able to diminish in an appreciable degree the appalling mortality among the new-born?

